# WallSense: Device-Free Indoor Localization Using Wall-Mounted UHF RFID Tags

**DOI:** 10.3390/s19010068

**Published:** 2018-12-25

**Authors:** Liang Ma, Meng Liu, Hongjun Wang, Yang Yang, Na Wang, Yajun Zhang

**Affiliations:** School of Information Science and Engineering, Shandong University, Qingdao 266237, China; millilitre@126.com (L.M.); liumengup@126.com (M.L.); nawang_vc2014@163.com (N.W.); yajunzhang369@163.com (Y.Z.)

**Keywords:** RFID, indoor localization, device-free, PSO, low cost

## Abstract

To achieve device-free indoor localization without the active participation of the users, this paper presents WallSense, a device-free indoor localization system based on off-the-shelf Radio RFID (Radio-Frequency Identification) equipment. The system deploys two orthogonal tag arrays in adjoining walls and uses the RSSI and phase information measured by RFID readers to localize the target. By differentiating the backscattered signal between adjacent tag pairs, WallSense is able to eliminate most undesirable factors and extract information directly related to the location of the target. By applying Particle Swarm Optimization (PSO) with a novel weighted fitness function and combining the localization result of two orthogonal tag arrays, the system is able to localize the target with high accuracy. Experiments show that the system is able to localize human target with 0.24 m median error. Also, WallSense has low deployment overhead and do not require the users to carry any devices.

## 1. Introduction

Currently a heated research topic, indoor localization has applications in health care [1,2], building automation [3], security [4], retail [5], entertainment [6], and assisted living [7,8]. The widely used outdoor positioning solution Global Positioning System (GPS) performs poorly indoors because the direct transmission path it requires is obstructed in indoor scenarios [9]. This gives rise to a series of research papers on indoor localization techniques and test methods [10].

Among those indoor localization approaches, device-free localization methods have more application potential as it doesn’t require users to carry any devices or tags [11]. Consider the scenario of elderly care, in which the location of the elderly has to be monitored. Using a device-based localization method would cause extra trouble to the elders as they have to remember to carry the device around. Some elders may have dementia and forget to carry the device. As a result, a device-free method would be highly desirable [12,13]. Video-based methods provide a good solution in public areas [14], but cannot be deployed in private room due to privacy issues [15,16,17]. RF-based device-free localization methods, however, do not require line-of-sight, preserves privacy, and do not have to be in direct contact with the users [11,12]. This makes it ideal in areas such as intruder detection [18], fitness tracking, elderly monitoring and law enforcement such as rescue missions [19].

However, current device-free localization methods either require costly customized hardware, or require a lot of site surveying, which is detrimental to the large-scale deployment of the system. In order to solve this problem, our goal is to build a device-free localization system that is low-cost, easy-to-deploy and have high scalability. In this paper, we present WallSense, a RFID-based device-free localization system built with Off-the-Shelf RFID readers and tags.

WallSense deploys arrays of passive RFID tags on adjoining walls as probes and measure the radio signal bouncing off the target. By deriving a propagation model and utilizing a technique that differentiates the signal between adjacent tags, WallSense is able to eliminate most undesirable factors and extract information directly related to the location of the target. A particle swarm optimization (PSO) algorithm is then applied to combine the location information provided by all the tag pairs using a weighted method and pinpoint the location of the target. To validate our propagation model and evaluate the positioning algorithm, we implemented WallSense using off-the-shelf Ultra High Frequency (UHF) RFID equipment and conducted a series of experiments. In our experiments, WallSense exhibited high accuracy and were robust against carrier frequency.

The rest of the article is organized as follows: Related works are introduced in Section 2. Technical background and the channel model applied is introduced in Section 3, and Section 4 gives a detailed account of the design of the localization system. The implementation and evaluation of the system are elaborated in Section 5 and Section 6 respectively. In the last section, a discussion concludes our work.

## 2. Related Works

Radio-frequency based indoor localization methods can be classified into two categories: device-based and device-free localization. Device-based localization systems require that the target carries an extra device that can communicate with other nodes [11]. The system locates the target by locating the device it carries. For example, pieces of baggage can be tracked by locating the RFID tags attached to them in an automatic sorting system [20,21] at airport terminals. Device-free methods, on the other hand, detects and locates the target by observing changes in the environment, without active participation on the targets’ side [22]. In 2007, the notion of device-free indoor localization was first defined by Moustafa Youssef et al. [11]. Since then, Device-free indoor localization methods have received much attention from researchers over the past decade. Among them, methods have been proposed based on radio frequency [12,19,23], optics [14,24], sound [25,26] and others like electrical field [27]. Over the years, researchers have proposed methods that realize RF-based device-free localization from many different approaches including radio grid, fingerprinting, RF backscatter and radar.

1. Methods based on radio-grid use multiple transceivers to form a radio grid and detecting the target by analyzing the attenuation between the links [28,29]. A training phase is often required to calibrate the parameters of the links. By combining with particle filters with RF tomographic imaging, researchers are able to track a time-varying group of (up to 4) persons with high accuracy [30,31]. On the other hand, however, these methods require densely distributed sensor nodes in small areas to get high accuracy, which lowers its application value.
2. Methods based on fingerprinting first construct a radio map with the target being at predetermined locations, then determine the location of the target by looking up the radio map [32,33]. TagTrack [34] deploys tags on the floor in a matrix and track human beings using K nearest neighbor (KNN) and Hidden Markov Model (HMM). Its average positioning accuracy is 0.7 m. Li et al. [23] proposed a scheme that boosts accuracy by improving Weighted-KNN (WKNN) algorithm. Through applying principal component analysis and successive cancellation, methods like SCPL are able to detect the number of subjects and simultaneously localize multiple subjects [31,35]. This approach provides reasonable localization accuracy with low hardware cost. However, constructing the radio map would require measuring the RSS values with the target being at a large number of predetermined locations, which is time-consuming and costly.
3. Methods based on RF backscatter observe the change in the environment by calculating the backscattered signal from reflective objects or passive tags [36,37]. This technique is common in RFID systems that use passive tags, in which tags attached to static objects in the area of interest serve as sensors. D-watch [38] used commodity RFID readers and tags to localize people in rich multipath environments by triangulating the directions that have the greatest power drops. The method has 16.5 cm median error. However, this method requires several antenna arrays to calculate the direction of incoming tag backscatter signals. Moreover, the method requires multiple Impinj GPIO adaptor and antenna hubs, which would introduce attenuation and limits the interrogation range of the system. Tadar [19] develops a high gain directional antenna and combines a multipath propagation model with HMM to realize through wall target tracking. Its median localization accuracy is 7.8 cm in the X dimension and 20 cm in the Y dimension. However, it involves using specifically designed hardware and requires calibration to remove the error caused by tag diversity to get a precise localization result. Also, Tadar requires the knowledge of the distribution of the observations when the object is at a certain location in order to calculate the emission probability of its HMM. Thus, Tadar is only suitable for small-scale through-wall localization applications.
4. Methods based on radar localize the target using radar methods [39,40]. Witrack [41] proposes a system based on Frequency-Modulated Continuous Wave (FMCW) that is capable of precise device-free localization and gesture recognition. The improved version [42] is able to localize multiple persons by applying successive cancellation. Its median localization accuracy is 10 to 13 cm. However, Witrack is built with a USRP device with high bandwidth and a specially designed T-shape antenna array, which limits the scale of its applications. Other radar-based systems have similar cost issues [43,44,45].

## 3. Channel Model of RFID backscatter

### 3.1. Technical Background

Consider a linear time-variant communication system that multiple transmitters transmit signals to a single receiver through a wireless channel. For a transmitted wireless signal denoted as *s*(*t*), the received signal can be expressed as:

(1) y(t)=h·s[t].

where $h=\alpha e^{j\delta }$ is a complex number known as the channel parameter [46]. α and δ are channel attenuation and phase rotation, respectively.

Another property of linear time-variant systems is used: when two sources *A* and *B* transmit simultaneously, the received signal can be represented as the combination of the signals from the two sources. This property is known as the rule of linear combination [47],

(2) $$y^{\prime }\left(t\right)=y_{A}\left(t\right)+y_{B}\left(t\right)$$

where $y_{A}\left(t\right)$ and $y_{B}\left(t\right)$ refer to the signal captured by the receiver when there is only *A* or *B* exists in the environment respectively.

In passive RFID systems, the communication is driven by the reader and the tag modulates the signal backscattered from its antenna to form a response. In common commercial readers, Received Signal Strength Indicator (RSSI) value and phase value are calculated after a tag Electronic Product Code (EPC) is demodulated successfully.

### 3.2. Channel Model

Now we consider the Line of Sight (LOS) path between a reader *R* and a tag *T*. In this situation, there are only two channels exists between reader *R* and tag *T*: forward channel and backward channel. Ideally, the channel parameters can be represented as [48]:

(3) $$h_{RT_{i}}=h_{T_{i}R}=\frac{1}{d_{RT_{i}}^{2}e^{j\theta_{RT_{i}}}},$$

where $d_{RT_{i}}$ is the distance between the Reader and the *i*-th tag. The $\theta_{RT_{i}}$ is the phase shift over the distance $d_{RT_{i}}$, which can be calculated given wavelength lambda of the carrier wave using the following formula:

(4) $$\theta_{RT_{i}}=2\pi \frac{d_{RT_{i}}}{\lambda }\;mod\;2\pi .$$

Due to geometric symmetry of the forward channel and the backscatter channel, it is easy to see that $d_{RT_{i}}$ = $d_{T_{i}R}$ and $h_{RT_{i}}$ = $h_{T_{i}R}$.

Denote the signal transmitted by the reader as $S_{0}$, the signal reflected from tag $T_{i}$ and received by the reader can be represented as

(5) $$S_{R_{i}}=S_{T_{i}}h_{T_{i}Tx}h_{T_{i}R}h_{RRx}.$$

However, in a real indoor environment, the signal is affected by multipath effect as there are lots of reflectors in the environment. First, consider the scenario that the tags are attached to the inner wall of a room and only static objects like chairs and walls exist in the environment. The tags and readers are all mounted on the walls so that people or other objects of interest do not obstruct the LOS path between the reader and the tags. Reflections caused by these objects can be treated as if they are from a single virtual reflection point *W* due to the principle of linear superimposing of signals [19].

Then the signal received by the tag can be represented as

(6) $$S_{T_{i}}=S_{RT_{i}}+S_{RWT_{i}}.$$

which is a combined signal of the LOS path and the signal reflected by the environment.

$S_{RWT_{i}}$ is given by:

(7) $$S_{RWT_{i}}=S_{0}h_{RTx}h_{RW}h_{w}h_{WT_{i}}h_{T_{i}Rx}.$$

In which $h_{RTx}$ is the transmitting parameter of reader antenna, $h_{RW}$ is the channel parameter of the channel between the reader antenna and virtual reflection point *W*. $h_{w}$ is the parameter introduced by reflecting at *W*, $h_{T_{i}Rx}$ is the receiving parameter of tag antenna. 

So, we have:

(8) $$\begin{array}{l} S_{T_{i}}=S_{RT_{i}}+S_{RWT_{i}}\\ \;\;=S_{0}\left(h_{RTx}h_{RT_{i}}h_{T_{i}Rx}+h_{RTx}h_{RW}h_{w}h_{WT_{i}}h_{T_{i}Rx}\right). \end{array}$$

**Figure 1:**
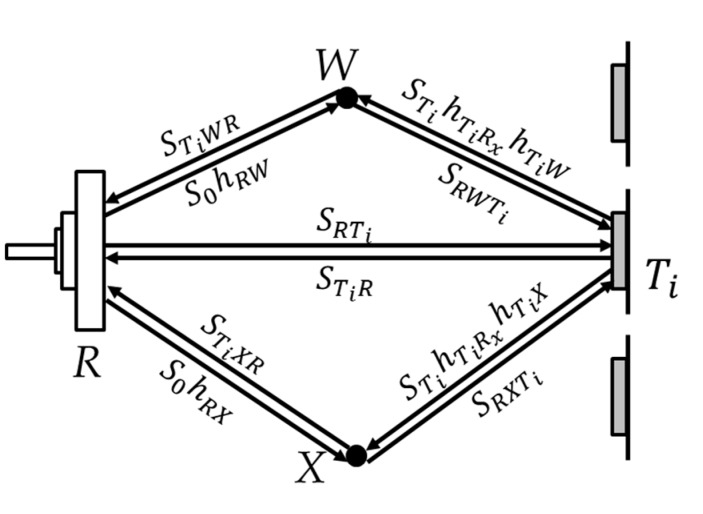
The signal propagation model.

As is shown in Figure 1, if a moving object *X* enters the area of interest, it will introduce a third signal path propagating from the reader antenna, reflected by object *X* then received by the tag antenna. In this scenario, the signal received by the tag is given by:

(9) $$S_{T_{i}}^{\prime }=S_{RT_{i}}+S_{RWT_{i}}+S_{RXT_{i}}$$

(10) $$S_{RXT_{i}}=S_{0}h_{RTx}h_{RX}h_{X}h_{XT_{i}}h_{T_{i}Rx}$$

(11) $$S_{R_{i}}^{\prime }=S_{T_{i}}^{\prime }\left(h_{T_{i}Tx}h_{T_{i}R}h_{RRx}+h_{T_{i}Tx}h_{T_{i}X}h_{X}h_{XR}h_{RRx}+h_{T_{i}Tx}h_{T_{i}W}h_{w}h_{WR}h_{RRx}\right),$$

where $S_{RXT_{i}}$ denotes the signal introduced by the moving object. $S_{R_{i}}^{\prime }$ denotes the signal reflected by tag $T_{i}$ and received by the reader.

## 4. Proposed Positioning Algorithm

### 4.1. Data Preprocessing

In order to remove the interfering parameters, we perform operations as follows:

First, by subtracting (5) from (11), we get

(12) $$\begin{array}{l} S_{R_{i}}^{\prime }-S_{R_{i}}=S_{T_{i}}^{\prime }\left(h_{T_{i}Tx}h_{T_{i}R}h_{RRx}+h_{T_{i}Tx}h_{T_{i}X}h_{X}h_{XR}h_{RRx}+h_{T_{i}Tx}h_{T_{i}W}h_{w}h_{WR}h_{RRx}\right)\\ \;\;\;-S_{T_{i}}(h_{T_{i}Tx}h_{T_{i}R}h_{RRx}+h_{T_{i}Tx}h_{T_{i}W}h_{w}h_{WR}h_{RRx}) \end{array}$$

where $S_{R_{i}}^{\prime }$ is the tag-reflected signal when object *X* is present, and $S_{R_{i}}$ is the tag-reflected signal when object *X* is absent.

Since the signal path that is introduced by the reflection of the surrounding environment and the target are much weaker than the LOS path, here we assume that the signal that is reflected twice during the round trip between the reader antenna and the tags can be negligible. Also, due to geometric symmetry, parameters like $h_{T_{i}R}\;$ is equal to its backward counterpart $h_{RT_{i}}$. Equation (12) is then simplified as:

(13) $$S_{R_{i}}^{\prime }-S_{R_{i}}=2S_{0}h_{RTx}h_{RX}h_{X}h_{XT_{i}}h_{T_{i}Rx}h_{T_{i}Tx}h_{T_{i}R}h_{RRx}$$

Divide the result among the neighboring tags, we get

(14) $$\frac{S_{R_{i+1}}^{\prime }-S_{R_{i+1}}}{S_{R_{i}}^{\prime }-S_{R_{i}}}=\frac{h_{XT_{i+1}}h_{T_{i+1}R}}{h_{XT_{i}}h_{T_{i}R}}$$

Here we assume that the tags are all of the same type and adjacent tags are close to each other. So that the differences of $h_{T_{i}Tx}$ of adjacent tags can be neglected. Since the locations of the readers and the tags are already known, $h_{T_{i}R}$ can be calculated using Equation (3), thus, only $h_{XT_{i}}$ component remains unknown. 

By definition, $h_{XT_{i}}$ is the loss of the *X* to $T_{i}$ path. It is a function of the distance between the tag and the moving object. It has the same form with $h_{T_{i}R}$. In light of this, $h_{XT_{i+1}}/h_{XT_{i}}$ can be expressed as:

(15) $$\frac{h_{XT_{i+1}}}{h_{XT_{i}}}=\frac{1}{{\left(\frac{d_{XT_{i}}}{d_{XT_{i+1}}}\right)}^{2}e^{j\left(\theta_{XT_{i}}-\theta_{XT_{i+1}}\right)}}.$$

Variable $\theta_{XT_{i}}$ is the phase shift over the distance between the moving object and the *i*-th tag. According to Equation (15), $h_{XT_{i+1}}/h_{XT_{i}}$ can also be expressed in the form of Rssi and phase value, and the phase value is:

(16) $$∆\theta_{i}=\theta_{XT_{i}}-\theta_{XT_{i+1}}$$

According to Equation (4), we have:

(17) $$∆d_{i}=d_{XT_{i}}-d_{XT_{i+1}}=\left(\frac{∆\theta_{i}}{2\pi }+n\right)\lambda ,\;\;\;\;\;\;\;n\in Z$$

where *n* is an integer introduced due to phase wrapping, $∆d_{i}$ is the difference between distances to the location of the target. To eliminate the ambiguity caused by *n*, a popular method is to deploy tags with a small interval. In this case, the range of $∆d_{i}$ must be smaller than the wavelength of the carrier wave to prevent phase ambiguity. For a tag array that is infinitely long, the range of $∆d_{i}$ is two times the interval between the tags. Therefore, the spacing of the tags should be less than half of the carrier wavelength. For each tag pair, $∆d_{i}$ is determined, thus we know that the location of the target is on a hyperbola for which $∆d_{i}$ is the focal distance and the locations of the two tags are the foci.

### 4.2. Particle Swarm Optimization

From Section 3.1 we know that the target location is on the hyperbolas with the tag pairs as their foci. The traditional method computes the target location by solving the points of intersection of the hyperbolas. However, this method not only has high computational costs, but also has low localization accuracy due to the low Signal to Noise Ratio (SNR) of backscattered signal.

Another approach to address the issue is by finding the position that most likely produces the set of ∆θ values observed. We use Particle swarm optimization (PSO) algorithm to solve this problem.

PSO algorithm is an efficient evolutionary computational technique for dealing with complex optimization problems. It is motivated by the behavior of a bird flock searching for food, in which birds tend to move towards the position with the most food that has been found by the flock. In each iteration of the algorithm, the basic component of the algorithm referred to as a particle, move toward the best performing particle and the best position found by the particle itself. After updating the positions of the particles, the evaluation of each particle is calculated, and the best positions are updated [49,50]. The particles will converge on the best position found by the particle swarm, thus solving the optimization problem. The algorithm is efficient and can easily benefit from multithread computational acceleration.

### 4.3. Positioning Using PSO Algorithm

WallSense treats the positioning problem as an optimization problem. By performing preprocessing, WallSense transforms RSSI and phase values to phase difference from the moving object to each tag pair. For each position in the area under surveillance, theoretical phase difference values of all the tag pairs can be calculated since the position of all the tags are already known. Thus, the positioning problem is transformed into an optimization problem in which the error between the measured phase difference values and the theoretical phase difference values is minimized. 

#### 4.3.1. Objective Function

For a set of measurements, WallSense first calculates the ${\hat{∆\theta }}_{i}$ values according to the processes described above. For a particle that has position value $X_{q}$, WallSense generates the theoretical $∆\theta_{i}\left(X_{q}\right)$ values according to the model. The output of the objective function is the Euclidean distance of the two set of ∆θ values.

Taking measurement noise of the reader into account, the signal measured by the reader when the target is not in the environment can be expressed as:

(18) $$S_{R_{i}}=\left(S_{RT_{i}}+S_{RWT_{i}}\right)h_{T_{i}}h_{T_{i}T_{x}}h_{T_{i}R}h_{RR_{x}}+\;\varepsilon ,\;\varepsilon \sim N\left(\mu ,\sigma^{2}\right).$$

In which ε represents measurement noise. The signal measured by the reader when the target is in the environment can be expressed as:

(19) $$S_{R_{i}}^{\prime }=\left(S_{RT_{i}}+S_{RWT_{i}}+S_{RXT_{i}}\right)h_{T_{i}}h_{T_{i}T_{x}}h_{T_{i}R}h_{RR_{x}}+\;\varepsilon^{\prime },\;\varepsilon^{\prime }\sim N\left(\mu ,\;\sigma^{2}\right).$$

Since all the measurements are taken with one reader, we assume the noise terms of all the measurements have the same parameters. By subtracting the signals measured without the target object in the area from the signal measured with the target object in the area, we extract the signal that transfers through the extra propagation path introduced by the target object. Subtracting (18) from (19), we get:

(20) $$S_{R_{i}}^{\prime }-S_{R_{i}}=S_{RXT_{i}R}+\;∆\varepsilon .$$

In which,

$$S_{RXT_{i}R}=S_{RXT_{i}}h_{T_{i}}h_{T_{i}T_{x}}h_{T_{i}R}h_{RR_{x}}\propto \frac{1}{d_{T_{i}R}^{2}},$$

$$∆\varepsilon =\varepsilon^{\prime }-\varepsilon ,\;∆\varepsilon \sim N\left(0,\;2\sigma^{2}\right).$$

The SNR of the *i*-th tag, $SNR_{i}$, can be expressed as:

(21) $$SNR_{i}=\;\frac{P\left(S_{RXT_{i}R}\right)}{P\left(∆\varepsilon \right)}\propto 1/d_{T_{i}R}^{4}.$$

Since the power of this signal is in reverse relation with the distance between the tag and the target object, signal backscattered from tags closer to the target object tend to have higher SNR.

Although the distance between the target and the tag pair is still unknown, for an array of tags that is uniformly arranged in a direct line, the tag pairs closer with the target object have smaller distance difference to the target, thus they also produce smaller ∆θ values. Thus, we weight the error of each tag pairs with a term $e^{-\left|{\hat{∆\theta }}_{i}\right|}$, to give more importance to the tag pairs that has smaller ∆θ values, as they are more probable to be closer to the target object.

(22) $$F\left(X_{q}\right)={\left\{\;\sum_{i=1}^{n-1}{\left[\left(∆\theta_{i}\left(X_{q}\right)-{\hat{∆\theta }}_{i}\right)\cdot \left(e^{-\left|{\hat{∆\theta }}_{i}\right|}\right)\right]}^{2}\right\}}^{1/2}$$

Note that $∆\theta_{i}\left(X_{q}\right)-{\hat{∆\theta }}_{i}$ is weighted with $e^{-\left|{\hat{∆\theta }}_{\;i}\right|}$, which is negatively correlated with the distance between the tag pairs and the moving object, thus reducing the influence of outlying tag pairs, which tend to have higher error because of the reflected signal is weaker.

#### 4.3.2. Swarm Initialization, Inertial Weight, Learning Factors and Population

On each dimension, WallSense initialize the positions of the particles randomly based on a uniform distribution defined in the area of interest.

The initial velocity of each particle is set to half of the distance between the particle and a random position in the area of interest. For each dimension d, the initial velocity of the *n*-th particle is:

(23) $${v\left(0\right)}_{n,d}=0.5\left(U\left(x_{min,d},x_{max,d}\right)-{x\left(0\right)}_{n,d}\right)$$

where ${x\left(0\right)}_{n,d}$ is the initial location of the *n*-th particle in dimension *d*, $x_{min,d}\;\mathrm{and}\;x_{max,d}$ are the lower and upper bound of the location of the particles in dimension *d*. To promote exploration in early optimization stages and reduce oscillation in later stages, WallSense uses a linearly decreasing inertial weight defined by [49]:

(24) $$w\left(t\right)=w_{0}-\left(w_{0}-w_{min}\right)\frac{t}{T_{max}}.$$

where $w_{0}$ is the starting value of inertial weight, $w_{min}$ is the desired minimum value of inertial weight and $T_{max}$ is the total number of iterations. $T_{max}$ is set to 100.

To get the best performance, WallSense used PSO algorithm and a training set to let the PSO learn the best learning factors for solving this positioning problem. 

The swarm population N is calculated according to the empirical formula [50]

(25) $$N=Int\left(10+2\sqrt{D}\right)$$

where *D* = 2 is the dimension of the problem and *Int*() is the integer part function.

#### 4.3.3. Enhancing Localization Accuracy and Robustness

**Figure 2:**
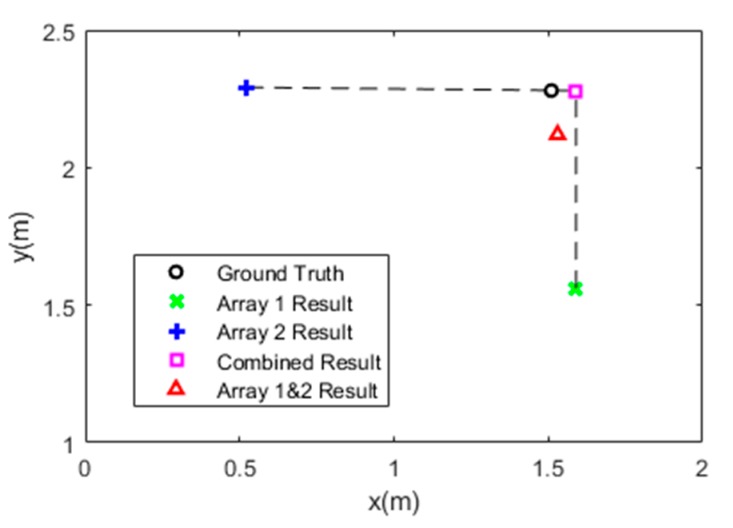
An example of using data from two arrays separately to improve localization result.

To further enhance the localization accuracy and robustness of the system, we adopted a technique to locate *x* axis and *y* axis of the target object separately. Because the location of the target is basically the intersection of all the parabolas defined by the tag pairs and $∆d_{i}$, one tag array tends to have high localization accuracy on the axis that is parallel to the tag array but have very low accuracy on the axis that is orthogonal to the tag array. We then used two separate tag arrays to locate *x* axis and *y* axis respectively. As is shown in Figure 2, the circle shows the ground truth. The accuracy difference of one array on the two axes can be very large. By discarding erroneous results and combining the accurate axis of the two arrays, this method can achieve higher accuracy compared to locating *x* axis and *y* axis using data from the two tag arrays at the same time. This is because when combining the data from the two tag arrays, the bad performance that one array has on the orthogonal axis would affect the good performance of the other array, resulting in worse overall performance.

### 4.4. Algorithm Summary

The discussion above has shed light on how to extract the reflection of the moving object as features and how to track the position of the moving object by using PSO algorithm. By putting them together, we get the following algorithm below:

Step 1: WallSense learns the backscatter signal $S_{R}^{\prime }$ that involves reflections from static objects in the environment with tags attached to the wall.

Step 2: When the object of interest is in the test environment, WallSense measures the backscatter signal $S_{R}^{\prime \prime }$ of each tag at each frame.

Step 3: WallSense calculates $\frac{S_{R_{i+1}}^{\prime }-S_{R_{i+1}}}{S_{R_{i}}^{\prime }-S_{R_{i}}}$.

Step 4: WallSense calculates $h_{XT_{i+1}}h_{T_{i+1}R}/h_{XT_{i}}h_{T_{i}R}$ according to Equation (14).

Step 5: WallSense calculates $h_{XT_{i+1}}/h_{XT_{i}}$ by removing $h_{T_{i+1}R}/h_{T_{i}R}$ according to the deployed locations of the antennas and tags.

Step 6: WallSense calculates $∆\theta_{i}$ according to Equation (15).

Step 7: The set of $∆\theta_{i}$ is fed into the PSO algorithm to determine the location of the target object. To boost accuracy and robustness, the objective value is weighted with $e^{-\left|{\hat{∆\theta }}_{\;i}\right|}$ for each tag pairs, and the *X* and *Y* coordinate are calculated separately with data collected from two orthogonal tag arrays.

## 5. Implementation

To evaluate the performance of WallSense in real world applications, we build a prototype of WallSense using COTS RFID reader and tags. 

### 5.1. Hardware

We use an Impinj Speedway R420 model [51] with manufacturer provided firmware and software. The reader is compatible with EPC Class 1 Generation 2 tags. It operates in 920 MHz~926 MHz band and provide an interface for acquiring Rssi and phase data for each successful identification. 

The inlay of the tags used in our experiment is AZ9662 with Higgs-3 chip [52]. Before the experiment, the tags are tested using Voyantic Tagformance Pro [53] to ensure that they have similar frequency response, thus eliminating the $h_{Ti}$ factor in (12). Tags that have bad performance are considered defective tags and are not used in our experiments. The Tags are arranged on foam boards for performance issues when attached directly on concrete walls. The spacing between adjacent tags is 15 cm.

The antennas used in our experiments are Laird S9028PCL circular polarization antennas [54] with 9dBi gain. The size of the antennas is 25.5 cm × 25.5 cm × 3.2 cm. The maximum read range of the RFID system equipped with this antenna is about 10~12 m.

We use a BOSCH GLM 30 laser rangefinder [55] to measure and calibrate the location of the antennas and tags. The location of the targets is measured with a tape measure with the help of a grid drawn on the floor.

### 5.2. Software 

Because the MultiReader software provided with the R420 readers does not provide phase information and cannot save more than a certain number of records, we developed a windowed software using Impinj Software Developer’s Kit (SDK) [56] to collect Rssi and phase data. The software is able to control readers that support the Low Lever Reader Protocol (LLRP) [57] through Ethernet cables and save the inventory history to a file. Localization algorithm is implemented in Matlab. The software and localization process run on a laptop with Intel I5-7300HQ CPU operating at 2.5 GHz and 8 GB memory. During the experiments, the read mode of the reader is set to MaxThroughput so that the reader dynamically adjusts the number of time slots in each frame based on the estimated tag population. Note that in this mode, the tags respond with FM0 coded bursts that have the highest data rate. This enables the reader to identify more than 200 tags per second.

## 6. Evaluation

**Figure 3:**
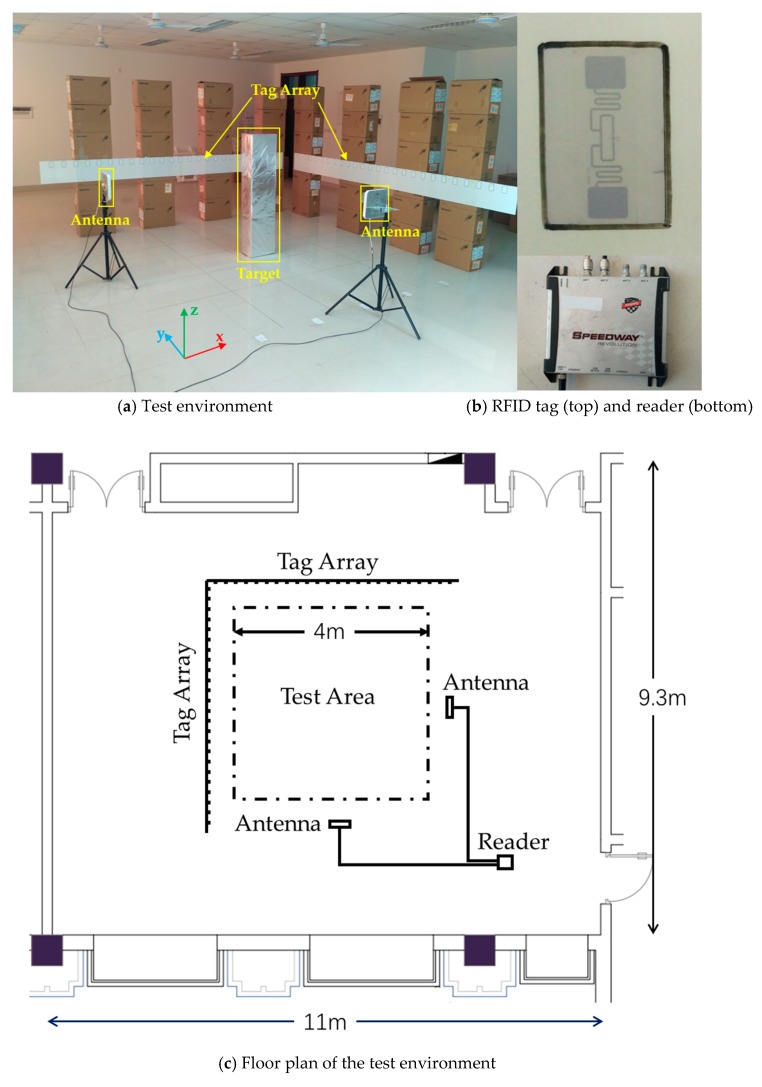
(**a**) Wall-mounted tag arrays and antennas in WallSense. (**b**) Tags are attached onto foamed plastic pads. The figure also shows the R420 reader used in our experiments. (**c**) Deployment layouts with the positions of tag arrays, antennas and test area marked.

In this section, the performance of WallSense is evaluated from aspects of localization accuracy, impact of distance to the tag array and impact of carrier frequency. The experiments are conducted in a large empty room of 11 m × 9.3 m × 3 m. The room has concrete wall, ceramic tile flooring and suspended ceiling with metal hangers and gypsum tiles. Metal ceiling fans are mounted on the ceiling. The experimental setup is shown in Figure 3. Two sets of tag arrays are deployed on the opposite wall of two antennas, covering a surveillance area of 4 m × 4 m. The “walls” that supports the tag arrays are stacks of cartons. Each set of tag arrays has 25 tags. The tags are deployed with 15 m spacing to prevent phase ambiguity in preprocessing. A reflective box of size 0.25 m × 0.37 m × 1.55 m and three volunteers of different height (1.65 m, 1.73 m, 1.78 m respectively) and build (51 kg, 60 kg, 68 kg respectively) served as the localization target in the experiments. The reflective box is a stack of 3 cartons coated with tin foil, which forms a highly reflective surface. Note that the coordinate of a target person is defined as the geometric center of his heels. The coordinates are measured with a tape measure and serve as the truth value in our experiments.

WallSense collects a set of RSSI and phase data of the tags when no target is in the area of interest as the reference. Note that this process only takes seconds and is very different from constructing a traditional radio map. We tested 120 uniformly distributed random positions in the area of interest. At each location, we collected the inventory data for 1 second and calculate the average RSSI and phase value to use as one sample. Of the three human targets, a total of 360 samples were collected.

### 6.1. Localization Accuracy

First, we evaluate the localization accuracy using one set of tag array and antenna. On each tag array, 21 tags are used to localize the target. Analyzing the localization result of both arrays, we found that the average localization accuracy of reflective box target is 0.18 m on the parallel axis, 1.60 m on the orthogonal axis. The average localization accuracy of human targets is 0.12 m on the parallel axis and 1.14 m on the orthogonal axis. Note that the localization error is defined as the distance between the localization result and the ground truth value.

**Figure 4:**
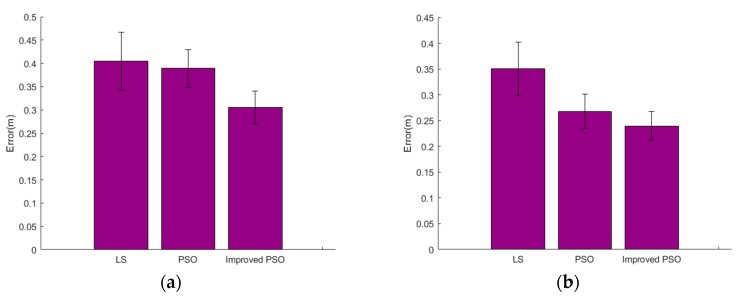
Localization accuracy of the proposed method, compared with Least Squares (LS)-based optimization. (**a**) Results with reflective box target. (**b**) Results with human target.

Figure 4 plots the localization accuracy of the system using two sets of tag arrays. The median localization error of the proposed method with human targets is 0.24 m, which is accurate considering the size of the human body. It can be seen on the figure that WallSense have high robustness by performing PSO twice. Using weighted PSO, WallSense yields a much better result than Least Squares (LS) based methods. The PSO algorithm converges in 30 iterations on average, which takes about several milliseconds.

We also evaluated the accuracy of WallSense when the reader was working at different frequencies. The results show that the localization accuracy remains stable with the change of carrier frequency. 

**Figure 5:**
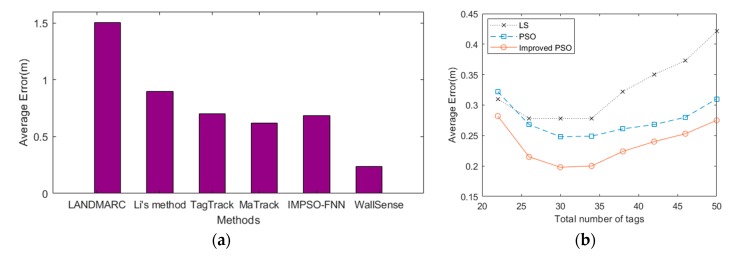
(**a**) The performance of WallSense compared with other device-free localization methods. (**b**) Impact of the number of deployed tags.

Figure 5 compares WallSense with other device-free indoor localization methods. Keep in mind that WallSense is a low-cost solution that only use off-the-shelf products and don’t require site surveying and carried devices.

### 6.2. Impact of the Number of Deployed Tags

To evaluate the impact of the number of deployed tags on the performance of WallSense, we compared the performance of the methods with the data of different number of tags in each tag array. The result is shown in Figure 5b.

For each method tested, the localization error first drops, then rises with increasing number of deployed tags. This is because when the number of deployed tags is below a threshold (about 15~19 tags each array), the information provided by the tags is not sufficient to rule out the influence of noise. When the number of deployed tags is beyond the threshold, the added tags are further away from the center of the tag array, these tags have low SNR and thus lower the localization accuracy of the system. 

**Figure 6:**
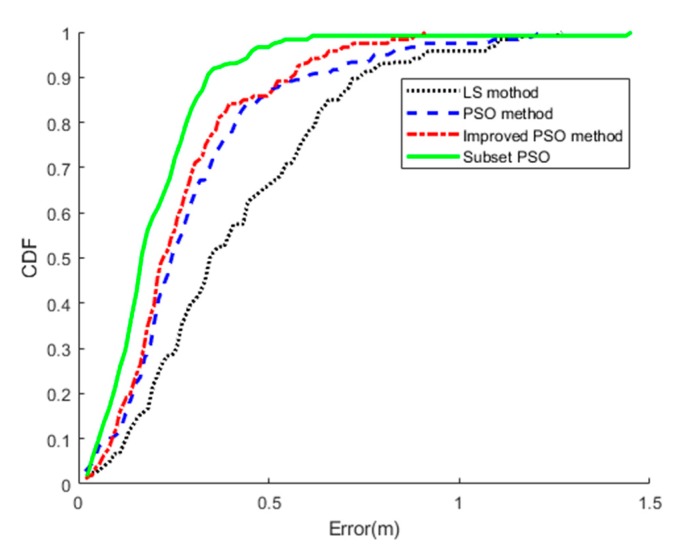
Comparison of the cumulative distribution function of the methods based on Particle Swarm Optimization (PSO). A total number of 50 tags are deployed. Subset PSO selects 34 of them for each particle.

However, it is possible to solve this problem by adding a tag-selection strategy for the particles. We implemented a strategy in WallSense that only picks a certain number of tags that are closest to a particle when calculating its fitness value. We found that the system is able to achieve a good overall result that is close to the result when an optimal number of tags are deployed. Using this method, WallSense can deploy as many tags as possible without worrying about lowering its accuracy. However, as is shown in Figure 6, there are rare cases in which the localization result become worse. Further studies into this subject are needed to improve the robustness of this method.

### 6.3. Cost

Low cost is a major advantage of our method compared to previous solutions. Utilizing only off-the-shelf readers and tags, WallSense is able to cover one room with two sets of tag array and two antennas. Due to the low cost of RFID tags (about $0.1 each), the tag arrays cost only several dollars in total. The antennas cost about $200. In our experiments, we adopt ImpinJ R420, a powerful reader which costs about $2000. But in real applications, this system can be deployed with cheaper readers such as the ThingMagic readers, which cost about $700. Furthermore, as most RFID readers support 4 or more antenna port, two adjacent rooms can share one reader. 

Although the location of the antennas and tags are required, the method requires no radio map and the target person doesn’t have to carry any additional devices.

## 7. Conclusions

In conclusion, WallSense is the first device-free indoor localization system that utilizes wall-mounted tag arrays to provide accurate indoor localization. By combining a PSO-based localization algorithm with a weighted objective function and deploying orthogonal tag arrays to locate *x* and *y* axis of the target location separately, WallSense is able to accurately and robustly locate human beings in an indoor setting. Comprehensive experiments prove that WallSense is an effective and promising indoor localization method that can strike a good balance between cost and performance.

## Abbreviations

EPC	Electronic Product Code
FMCW	Frequency-Modulate Continuous Wave
GPS	Global Positioning System
HMM	Hidden Markov Model
KNN	K Nearest Neighbor
LOS	Line of Sight
LS	Least Squares
PSO	Particle Swarm Optimization
RFID	Radio-Frequency Identification
RSSI	Received Signal Strength Indicator
SNR	Signal to Noise Ratio
SDK	Software Developer’s Kit
UHF	Ultra-High Frequency
USRP	Universal Software Radio Peripheral
WKNN	Weighted KNN
